# Menstrual cups and sanitary pads to reduce school attrition, and sexually transmitted and reproductive tract infections: a cluster randomised controlled feasibility study in rural Western Kenya

**DOI:** 10.1136/bmjopen-2016-013229

**Published:** 2016-11-23

**Authors:** Penelope A Phillips-Howard, Elizabeth Nyothach, Feiko O ter Kuile, Jackton Omoto, Duolao Wang, Clement Zeh, Clayton Onyango, Linda Mason, Kelly T Alexander, Frank O Odhiambo, Alie Eleveld, Aisha Mohammed, Anna M van Eijk, Rhiannon Tudor Edwards, John Vulule, Brian Faragher, Kayla F Laserson

**Affiliations:** 1 Department of Clinical Sciences, Liverpool School of Tropical Medicine (LSTM), UK; 2 Centre for Global Health Research, Kenya Medical Research Institute (KEMRI), Kisumu, Kenya; 3 Siaya District Hospital, Ministry of Health, Siaya, Kenya; 4 Centers for Disease Control and Prevention (CDC)-Kenya, Kisumu, Kenya; 5 Safe Water and AIDS Project (SWAP), Kisumu, Kenya; 6 Division of Reproductive Health, Ministry of Public Health and Sanitation, Nairobi, Kenya; 7 Centre for Economics and Policy in Health, Bangor University, UK; 8 Division of Global Health Protection, Center for Global Health, Centers for Disease Control and Prevention, Atlanta, Georgia, USA

**Keywords:** menstrual hygiene management, adolescent, sexual and reproductive health, sexually transmitted infections, reproductive tract infections, menstrual cups

## Abstract

**Objectives:**

Conduct a feasibility study on the effect of menstrual hygiene on schoolgirls' school and health (reproductive/sexual) outcomes.

**Design:**

3-arm single-site open cluster randomised controlled pilot study.

**Setting:**

30 primary schools in rural western Kenya, within a Health and Demographic Surveillance System.

**Participants:**

Primary schoolgirls 14–16 years, experienced 3 menses, no precluding disability, and resident in the study area.

**Interventions:**

1 insertable menstrual cup, or monthly sanitary pads, against ‘usual practice’ control. All participants received puberty education preintervention, and hand wash soap during intervention. Schools received hand wash soap.

**Primary and secondary outcome measures:**

Primary: school attrition (drop-out, absence); secondary: sexually transmitted infection (STI) (*Trichomonas vaginalis*, *Chlamydia trachomatis*, *Neisseria gonorrhoea*), reproductive tract infection (RTI) (bacterial vaginosis, *Candida albicans*); safety: toxic shock syndrome, vaginal *Staphylococcus aureus*.

**Results:**

Of 751 girls enrolled 644 were followed-up for a median of 10.9 months. Cups or pads did not reduce school dropout risk (control=8.0%, cups=11.2%, pads=10.2%). Self-reported absence was rarely reported and not assessable. Prevalence of STIs in the end-of-study survey among controls was 7.7% versus 4.2% in the cups arm (adjusted prevalence ratio (aPR) 0.48, 0.24 to 0.96, p=0.039), 4.5% with pads (aPR=0.62; 0.37 to 1.03, p=0.063), and 4.3% with cups and pads pooled (aPR=0.54, 0.34 to 0.87, p=0.012). RTI prevalence was 21.5%, 28.5% and 26.9% among cup, pad and control arms, 71% of which were bacterial vaginosis, with a prevalence of 14.6%, 19.8% and 20.5%, per arm, respectively. Bacterial vaginosis was less prevalent in the cups (12.9%) compared with pads (20.3%, aPR=0.65, 0.44 to 0.97, p=0.034) and control (19.2%, aPR=0.67, 0.43 to 1.04, p=0.075) arm girls enrolled for 9 months or longer. No adverse events were identified.

**Conclusions:**

Provision of menstrual cups and sanitary pads for ∼1 school-year was associated with a lower STI risk, and cups with a lower bacterial vaginosis risk, but there was no association with school dropout. A large-scale trial on menstrual cups is warranted.

**Trial registration:**

ISRCTN17486946; Results

**Strengths and limitations of this study**

- This is the first cluster randomised controlled study of menstrual products examining the effect of interventions on dropout, sexually transmitted infection and reproductive tract infection among schoolgirls.
- Follow-up of participants longitudinally for 9 months or more demonstrated the importance of product familiarisation before measureable effects were generated.
- Participant follow-up back to homes differentiated migration (loss to follow-up) from school dropout.
- Frequent follow-up to evaluate use and safety may have positively influenced participants' attitudes to attending school, affecting school outcomes, including in the control arm.
- Studies on menstruation in rural African primary schoolgirls are compromised by the increasingly younger age girls' complete school, with fewer girls reaching menarche during primary school.

## Introduction

Poor menstrual hygiene management (MHM) affects the dignity, health and well-being of schoolgirls in low and middle income countries (LMIC), and requires a range of Water, Sanitation, and Hygiene (WASH) interventions.^1^ ^2^ Many girls in LMIC cannot afford sanitary pads and resort to impromptu items like old cloth or cotton wool, or use a minimal number of pads, which leak, smell and chafe, causing them stigma, shame and discomfort.^3–6^ The use of unhygienic cloths for menstruation is associated with reproductive tract infection (RTI) symptoms, requiring verification.^7–9^ Qualitative studies show that poor MHM reduces a girl's ability to engage in class and may cause school absence and dropout.^3–5^ ^10^ School absence contributes to girls' life-chances by lowering performance, increasing grade repetition, pregnancy risk and dropout.^11^ However, no strong association has been found between absence and menstrual intervention to date,^10^ ^12–16^ and none have examined school dropout. Remaining in school acts as a ‘social vaccine’,^17^ protecting girls against sexually transmitted infections (STIs) and reproductive harms.^11^ ^18^ ^19^

Evidence is accruing that young adolescent girls engage in transactional sex to obtain sanitary pads.^3^ ^20–24^ In western Kenya, where HIV incidence rises sharply among adolescent girls,^22^ 10% of girls 15 years or younger admitted having transactional sex to obtain money to buy pads.^24^ Such behaviour can contribute to girls' exposure to STI,^25^ HIV,^22^ pregnancy and subsequent school dropout.^23^ Further, should provision of hygienic menstrual products affect the prevalence of bacterial vaginosis,^7–9^ this could impact on girls' susceptibility to STI,^26–29^ and HIV.^30^ ^31^ While two exploratory controlled studies providing pads and menstrual cups to Ghanaian and Nepalese schoolgirls, respectively, demonstrated marginal effect on school absence, they did not examine STI or RTI outcomes.^10^ ^14^ Menstrual cups have also been tested among women, showing women's increased preference for use over their usual practice, and no safety issues.^32–35^ We conducted a feasibility study to examine the acceptability and safety of menstrual cups and sanitary pads among primary schoolgirls in rural western Kenya and to obtain preliminary estimates of the effect sizes on a range of potential primary outcomes including school dropout, STIs and RTIs to support the design of a subsequent larger trial. The results of nested studies on study girls' menstrual needs and perceived benefits of using menstrual cups and sanitary pads are reported elsewhere.^3^ ^36–39^

## Methods

### Study design and participants

This was a three-arm, open-label, cluster randomised controlled feasibility study in 30 primary schools located in a rural area under a continuous Health and Demographic Surveillance System HDSS,health and demographic surveillance system (HDSS) in Gem District in Siaya County in western Kenya.^40^ In this area, girls have little access to hygienic menstrual products,^3^ ^23^ ^24^ ^36^ and are frequently exposed to sexual harms,^3^ ^22–24^ reflected in the rapid increase in HIV and herpes simplex virus type 2 (HSV-2) prevalence in girls between 13 and 18 years of age from ∼1% to 12.8% and 9% to 40%, respectively.^22^

There were 71 primary schools within the HDSS area in Gem District; 62 agreed to participate,^36^ five of which were ineligible (enrolled in another study, or missed eligible class years). Of the 57 remaining schools, 30 fulfilled the WASH eligibility criteria consisting of a separate girls' toilet block present, water present at spot-check, and a pupil–latrine ratio<70:1.

Schoolgirls were eligible if they were 14–16 years old, had no precluding disability, had experienced at least three menses, were resident for at least 4 months in the study area, and provided written assent (schoolgirl) and consent (parents/caregivers). Girls who were reported to be pregnant or were with visible pregnancies were excluded.

A sample size of 185 girls per arm from a population of 3165 girls was estimated to provide 5% precision of school dropout (primary outcome) if this occurred at 15% in the control arm. To allow for a design effect of 1.25 and 7.5% loss to follow-up, 250 girls (10 schools with an average of 25 girls per school) were scheduled for recruitment per arm. This same sample size would provide 4.6% and 4.2% precision if school dropout was 12.5% or 10%, respectively, in the intervention groups.

### Randomisation and blinding

The 30 eligible schools were matched in triplicate by the trial statistician based on the pupil–latrine ratio obtained from a baseline WASH study.^36^ Prior to a community randomisation ceremony, attended by all head teachers and District Education Officers (DEO), allocation assignments were sealed in three identical opaque envelopes by a non-involved administrator. During the ceremony, 10 sets of three head teachers, one for each school in a matched triplet, simultaneously picked and displayed one of three colour balls from an opaque bag. After all balls were chosen, a DEO opened the three envelopes to reveal the colour-allocated assignment. Following randomisation, participants and study nurses were made aware of the allocation, but allocation was concealed from the laboratory staff and the trial statistician until the database was locked.

### Procedures

Meetings were held in the 30 study schools with parents/caregivers, target-aged girls, and teachers to discuss the study and answer questions. Girls' homes were visited to provide information sheets, discuss the study with parents, and obtain parents' written consent. Information on geolocation of girls' residence was obtained from the HDSS database. Class meetings with girls described the study, provided information sheets and encouraged and answered questions, before obtaining participants' written assent.

At enrolment, individual participant's baseline characteristics were gathered through nurse interview and behavioural survey tools.^24^ Well-being was assessed using the Pediatric Quality of Life Inventory (PEDSQL) instrument.^41^ Household-level characteristics and socioeconomic status (SES) were obtained using the existing HDSS data.^42^ School-level characteristics on size and WASH were collected through unannounced spot-checks at baseline, and twice per term over the study.^36^ Participants were provided with adapted monthly calendars,^14^ to self-record daily school attendance and menstruation. This documented which days girls had their menstruation and which days they were absent from school. Calendars were formatted as a teleform; data was captured from weekly scanning of images to the local database, and then extracted to the raw database. Health facilities in the study area were visited to explain the study, toxic shock syndrome (TSS) symptoms and the process of tertiary care referral, and the researcher's contact details given. Communication was established with district hospitals for suspected TSS admissions. Participants, families, and schools received written information about the study, TSS symptoms, how to contact the study staff, and health facility referral.

Kenyan registered study nurses employed full time during the study visited schools weekly to follow-up girls and assess any safety issues. At each scheduled follow-up, nurses documented menstruation, intervention use, adverse events, and any problems on a one-to-one basis. Nurses physically checked girls' cups to ascertain use (by cup colour change), damage, or loss. Girls were referred to the district hospital gynaecologist for clinical assessment if required. The trained nurses instructed girls to self-complete a vaginal swab for *Staphylococcus aureus*,^43^ to evaluate vaginal colonisation, and positives were retested to evaluate the prevalence of TSS toxin production. A random sample of 35 cups was obtained to evaluate *Escherichia coli* growth. HDSS census and health clinic record reviews were conducted to assess any deaths, TSS, or other severe adverse events among participants.

Homes of all girls absent for a term were visited to document reasons for non-attendance, whether the participant had dropped out of school or was temporarily absent (eg, due to illness) or had migrated and when, and whether they were pregnant. If pregnant, information was collected to establish the estimated delivery time (ie, the dates of last menstrual period or conception, if known) and the birth outcome if delivery had taken place. This was conducted by community health workers in each village trained as ‘village recorders’ (VR) within the HDSS. Lists of absent girls and the geolocation of their residence were forwarded to each VR for follow-up. Participant dropout was identified through a computer-generated list of girls absent from nurse screening each term. Nurses verified with teaching staff if the listed participants did not attend school the previous term. If girls failed to attend a scheduled nurse screening visit but were reported as present in the school, an alternate screening visit date was arranged, or if the girl refused to attend a screening visit it was explored whether the girl intended to withdraw from the study. If girls were found to be absent from the school and if teachers confirmed non-attendance or did not know, girls were scheduled to be visited at home by a VR. All girls were visited at home at the end of the study to validate earlier findings, plus capture any missed dropouts, pregnancies, and pregnancy outcomes. At the end of the study, RTI/STI vaginal swabs were taken by girls with training and assistance from study nurses in a private room at school and girls' symptoms documented.^25^ Specimen were processed at KEMRI laboratories using AMPLICOR PCR for *Neisseria gonorrhoeae* and *Chlamydia trachomatis* detection; in pouch microscopy for *Trichomonas vaginalis* and *Candida albicans*, and Gram stain for bacterial vaginosis, and the Nugent Score (7–10) counted for *Gardnerella, Lactobacillus,* and *Mobiluncus.*^43^

### Interventions

All girls within the study schools who fulfilled the eligibility criteria at the start of the study were enrolled. If girls had not yet reached menarche, but otherwise fulfilled all other entry criteria, they were eligible for enrolment during the course of the study as soon as they started menstruating. At enrolment, all participants in each arm received puberty and hygiene training; girls in the cups and pads arms received menstrual product-specific training from study nurses after enrolment. Girls attending cup-allocated schools were each provided one menstrual cup (Mooncup (Mooncup, Brighton, UK), size B; see online supplementary figure S1) with written and verbal instructions on cup insertion and cleaning. Menstrual cups are reusable bell-shaped receptacles which collect ∼30 mL of menstrual blood when inserted into the vaginal canal.^44^ Emptying is required every 4–8 hours depending on menstrual flow. Once emptied can be immediately reinserted, with cleansing by boiling occurring at the end of a cycle. The Mooncup used in this study was sourced from the UK. It is made of high-grade medical silicone which is safe, durable, and pliable, and the material is hypoallergenic with no open pores or edges to harbour bacteria. They are manufactured in the UK to ISO 13485:2003 standards, and have approval from the US Food and Drug Administration. Mooncups are available for sale in Kenya, marketing at a cost of ∼$25 each in 2012. Girls in pad-allocated schools received 16 Always sanitary pads (2 packs per month) and corresponding instructions. Always sanitary pads are available in Kenya and were sourced locally in Kisumu for ∼$1 per pack in 2012. Girls attending control schools continued their usual practice. All girls received hand-washing soap, and pencils for calendar completion. All schools were provided with detergent soap each month for hand washing.

10.1136/bmjopen-2016-013229.supp1 Supplementary data

### Outcomes

The primary outcomes were school dropout, defined as non-attendance for one term with no return to school; and days' absence, defined as self-reported days absent per 100 schooldays (excluding weekends, holidays, strikes, etc). Prespecified key secondary outcomes included the prevalence of confirmed STIs (*T. vaginalis*, *C. trachomatis*, and *N. gonorrhoeae*) and RTIs (bacterial vaginosis and *C. albicans*) during the end line survey.

The main safety outcomes were the incidence of TSS among girls provided menstrual cups, and the prevalence of vaginal *S. aureus*.

### Statistical analysis

The primary analysis was in the intention-to-treat population, defined as eligible girls receiving the intervention per allocation group. For binary outcomes, we compared treatment groups with unadjusted and multivariate log-binomial Generalised Estimating Equations (GEE) models with the school as the cluster variable. Results were expressed as the risk ratio and 95% CI. For continuous responses, we used unadjusted and multivariate linear regression models using a normal distribution and identity link functions, and expressed results as the mean difference (95% CI). An independent covariance structure with a robust variance estimator was used for GEE models. Prespecified covariates included girls' baseline age, SES and self-reported sexual intercourse. Age at enrolment and age at menarche were documented as integers in years as girls did not know their dates of birth, with the majority increasing their annual age at each January school enrolment. Age was collapsed into 14 years and 15–16 years. SES index was calculated using a weighted average using multiple correspondence analyses (MCA).^45^ MCA indicators were generated from biennial household surveys in the HDSS, documenting the occupation of household head, primary source of drinking water, use of cooking fuel, in-house assets (eg, lantern lamp, sofa, bicycle, radio and television) and livestock (poultry, pigs, donkey cattle, sheep and goats).^46^ The SES of households for each participant was ranked into quintiles. This was collapsed into a dichotomous variable of poorest (40%) and less poor (60%). Two absence rates were created (a) days absence per 100 viable schooldays (ie, excluding weekends, and school closure days including holidays, school strikes, etc) and (b) days absent during period per 100 menstruating viable schooldays. Missing values for SES and reported sexual intercourse were imputed. Intracluster correlation coefficients (ICC) were calculated for primary outcomes. To determine the effect of duration on the treatment effect, secondary stratified analysis was conducted by the duration that girls were enrolled in the study. This was calculated from the date intervention provided to the date of the outcome measured. These were then collapsed into two categorical time thresholds, duration of follow-up at least 9 months (yes/no), and duration of follow-up for at least 12 months (yes/no). Finally, we compared the effects of cups among girls whose cup was observed by nurses to change colour, against those with no colour change and against controls. Analysis was conducted in SAS V.9.3 and SPSS V.22. No adjustment was made for multiplicity.

## Results

**Figure 1:**
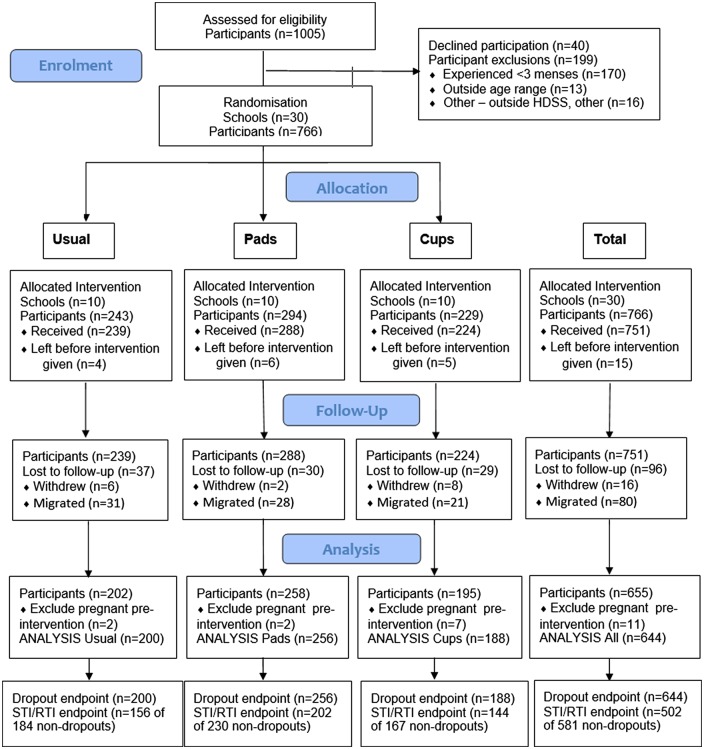
Participant flow diagram. HDSS, health and demographic surveillance system; RTI, reproductive tract infection; STI, sexually transmitted infection.

Girls were enrolled from 15 August 2012 to 27 August 2013 and followed until 21 November 2013. No schools withdrew from the study. Of 1005 girls in eligible classes, 199 (19.8%) did not fulfil the eligibility criteria, 40 (5.0%) eligible girls refused, and 15 (1.8%) migrated prior to the start of the intervention (figure 1). Of the remaining 751 receiving the intervention, 11 were excluded from analysis having been pregnant prior to the start of the intervention, and 96 (12.8%) were lost-to-follow-up (16 withdrew and 80 migrated). Loss-to-follow-up did not differ across groups. Girls were followed for a median (IQR) of 10.9 (6.1–12.5) months, 66.1% for 9 m or more (see online supplementary table S1). Overall, 644 girls in 30 schools (cups=188, pads=256, and control=200 girls) contributed to the analysis for dropout, and 502 to the STIs and RTIs analyses.

### Baseline characteristics

The mean (SD) age at enrolment was 14.6 (0.7) years. At baseline, 82.6% reported they had never used pads. The baseline characteristics were well distributed with the exception of the proportion of girls who reported having experienced sexual intercourse, which was higher in the cups (31.7%) compared with the pads (23.3%) and control (24.2%) arms (table 1).

**Table 1:** Demographic, menstrual and sexual/reproductive characteristics of study population at baseline (N=644)

Group	Characteristics*	Statistics/category	Control (%) (N=200)	Pads (%) (N=256)	Cups (%) (N=188)	Total (%) (N=644)
Sociodemographic	Grade at enrolment	Mean (SD)	6.8 (0.8)	6.8 (0.8)	6.7 (0.8)	6.8 (0.8)
	Age in years at enrolment	Mean (SD)	14.6 (0.7)	14.5 (0.7)	14.6 (0.7)	14.6 (0.7)
	Socioeconomic status†	n	171	216	156	543
		Poorest	43 (25.1%)	29 (13.4%)	22 (14.1%)	94 (17.3%)
	Lives with mother	n	194	249	183	626
		Yes	143 (73.7%)	166 (66.7%)	121 (66.1%)	430 (68.7%)
	Well-being (PEDSQL)	n	199	251	182	632
		Mean (SD)	409.4 (103.1)	436.5 (121.4)	420.3 (123.8)	423.3 (117.0)
Menstrual	Age in years at menarche	Mean (SD)	13.6 (0.8)	13.7 (0.8)	13.5 (1.0)	13.6 (0.9)
	Number of days menses	Mean (SD)	3.7 (1.2)	3.9 (1.3)	3.7 (1.5)	3.8 (1.3)
	Experience heavy periods	n	200	256	188	644
		Yes	41 (20.5%)	68 (26.6%)	39 (20.7%)	148 (23.0%)
	Experience period cramps	n	200	256	188	644
		Yes	129 (64.5%)	165 (64.5%)	115 (61.2%)	409 (63.5%)
	Report have ever used pads	n	200	256	188	644
		Yes	168 (84.0%)	198 (77.3%)	166 (88.3%)	532 (82.6%)
Sexual/reproductive	Report have ever had sex‡	n	194	249	183	626
		Yes	47 (24.2%)	58 (23.3%)	58 (31.7%)	163 (26.0%)
	Report have ever been pregnant	n	194	249	183	626
		Yes	0 (0%)	2 (0.8%)	2 (1.1%)	4 (0.6%)
	Report being married	n	194	249	183	626
		Yes	3 (1.5%)	4 (1.6%)	5 (2.7%)	12 (1.9%)

*Characteristics reported by participants at baseline survey.

†Poorest: lowest two quintiles; less poor: upper three quintiles of socioeconomic status index score computed by multiple correspondence analysis.

‡Ever had sex includes those reporting having had sexual intercourse, including those reporting tricked or forced to have sexual intercourse.

### Dropout

**Figure 2:**
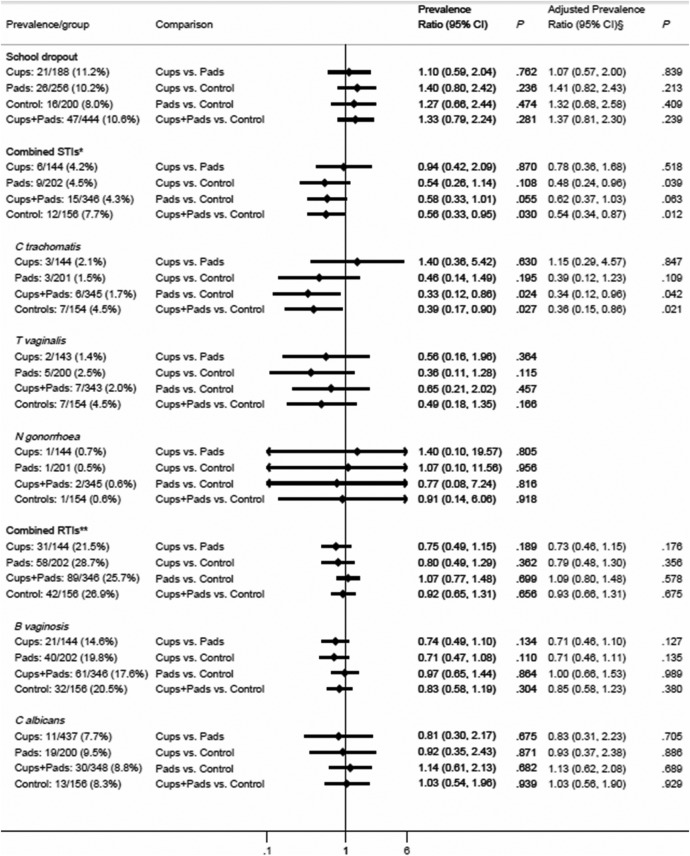
Impact of pads and cups on school dropout, STI and RTI. §Adjusted for age, socioeconomic status, and reported sexual activity (had sexual intercourse) at start of study. *Composite of STIs at end line (presence of either *C trachomatis*, *T vaginalis*, or *N gonorrhoea*). **Composite RTIs tested at end line (presence of either B vaginosis or *C albicans*). B vaginosis, bacterial vaginosis; *C albicans*, *Candida albicans*; *C trachomatis*, *Chlamydia trachomatis*; *N gonorrhoea*, *Neisseria gonorrhoea*; RTI, reproductive tract infection; STI, sexually transmitted infection; *T vaginalis*, *Trichomonas vaginalis*.

**Figure 3:**
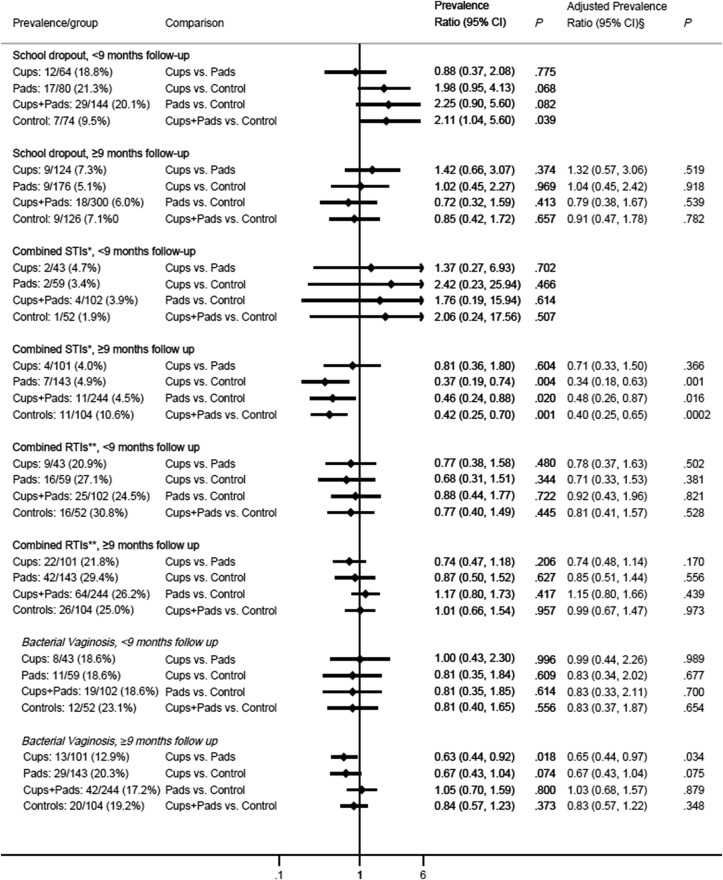
Impact of pads and cups on school dropout, sexually transmitted infections (STIs) and reproductive tract infections (RTIs) by 9 months duration. §Adjusted for age, socioeconomic status, and reported sexual activity (had sexual intercourse) at start of study. *Composite of STIs at end line (presence of either *Chlamydia trachomatis*, *Trichomonas vaginalis*, or *Neisseria gonorrhoea*). **Composite RTIs tested at end line (presence of either bacterial vaginosis or *Candida albicans*).

The cumulative risk of dropout by the end of follow-up was 11.2%, 10.2%, and 8.0% in the cups, pads, and control arms, respectively, and was mainly pregnancy-related (see online supplementary table S2) and did not differ significantly by arms overall (figure 2), or when stratified by duration (figure 3). The ICC for school dropout was 0·0084.

### Absence

A total of 5971 monthly calendars were submitted as completed by the participants during the study, with a median of 10 (IQR 6–12) calendars completed per participant. Self-reported school absence was very rarely reported (0.4 [2.0] days per 100 of all [menstruating] school days) precluding analysis.

### Sexually transmitted infections

The prevalence of all STIs at the end line survey was 7.7% in the control arm versus 4.3% in the pooled cups+pads arms (adjusted prevalence ratio (aPR) 0.54, 95% CI 0.34 to 0.87, p=0.012) and 4.2% in the cups arm (aPR=0.48, 0.24 to 0.96, p=0.04) and 4.5% in the pads arm (aPR=0.62, 0.37 to 1.03, p=0.06) (figure 2). The difference reflected lower prevalence of infection with *C. trachomatis* (prevalence ratio (PR) 0.39, 0.17 to 0.90, p=0.027) and *T. vaginalis* (PR=0.49, 0.18 to 1.35, p=0.17), but not *N. gonorrhoea* (PR=0.91, 0.14 to 6.06, p=0.92). Further analysis by duration since enrolment showed the greatest impact was among girls who had been exposed to the intervention for at least 9 months (figure 3) or 12 months (see online supplementary figure S2). The ICC for combined STI was 0.0191.

### Reproductive tract infections

The prevalence of RTIs (bacterial vaginosis or *C. albicans*) was 21.5%, 28.7% and 26.9% among cup, pad and controls arms, respectively (figure 2). Bacterial vaginosis comprised 71% of RTIs and was prevalent at 20.5% in the control arm. This was lower (not significant) in the cups arm (14.6%, PR=0.71, 0.47 to 1.08, p=0.11), but not the pads arm (19.8%, PR=0.97, 0.65 to 1.44, p=0.86; figure 2). Prevalence differences were again greatest among girls who had received the intervention for at least 9 months (control=19.2%, cups=12.9%, pads=20.3%) (figure 3), with a significantly lower prevalence in the cups compared with pads and control arms pooled (12.9% vs 19.8%, PR=0.65; 0.46 to 0.93; p=0.018). The ICC for combined RTI was 0·0031.

Outcomes among girls with no observed cup colour change were no different from controls (see online supplementary table S3).

### Harms

No serious adverse events were observed and no TSS cases reported (table 2). The prevalence of vaginal *S. aureus* was 9.6%, 11.2% and 11.3% in the cups, pads and control arms, respectively (table 2). Toxic shock syndrome toxin-1 (TSST-1) was detected in 2 of 10 *S. aureus* positive vaginal isolates; both were pad users and were healthy at follow-up (table 2). *E. coli* was grown on 37% (13 of 35) in-use sampled cups; this was in 53% (9 of 17) cups of newer (<6 m) users, versus 22.2% (4 of 18) in cups used for 6 m or longer, with no growth detected in six cups used for longer than 9 months.

**Table 2:** Safety outcomes

	Cups (N=188)	Pads (N=256)	Controls (N=200)	Total (N=644)
All severe adverse events				
Deaths recorded through HDSS	0	0	0	0
Participant identified to have symptoms of toxic shock syndrome	0	0	0	0
Visited health facility for toxic shock syndrome*	0	0	0	0
Other participant safety outcomes				
Referred for gynaecological check-up for heavy periods†	5/188 (2.7%)	5/256 (2.0%)	0/186 (0.0%)	10/644 (1.6%)
Prevalence of *Staphylococcus aureus* during follow-up	17/177 (9.6%)	27/214 (11.2%)	21/186 (11.3%)	65/604 (10.8%)
Prevalence of *S. aureus* at second survey of positives	4/12 (25.0%)	3/17 (17.6%)	3/16 (18.8%)	10/49 (20.4%)
Presence of toxic shock syndrome-1 toxin in *S. aureus* positive samples in second survey of positives	0/4	2/3	0/3	2/10

*Registries of all health facilities reviewed.

†Eight (four cup, four pad) of the 10 cases had recorded heavy periods at baseline.

HDSS, Health and Demographic Surveillance System of all homesteads.

## Discussion

To the best of our knowledge, this is the first intervention study to compare the feasibility and potential impact of menstrual cups and sanitary pads on schoolgirls' sexual and reproductive health and school dropout in sub-Saharan Africa (SSA). Both products were associated with a lower prevalence of STIs, *C. trachomatis* and *T. vaginalis* in particular. Given that girls in this area of Kenya are vulnerable to coercive sex to obtain necessities such as pads,^3^ ^4^ ^23^ ^24^ a beneficial impact on STIs after cup or pad provision is plausible. Menstrual cups were also associated with lower bacterial vaginosis prevalence among girls using them for at least 9 months. This is likely due to the fact that cups do not disrupt the vaginal flora or pH,^33^ while unhygienic cloth use has been associated with bacterial vaginosis in Tanzanian,^47^ and Indian women.^7^ The lack of an effect of pads on bacterial vaginosis may reflect girls obligation to share with others, leading to prolonged use of the few pads they retain;^37^ as girls reported ‘overstaying’ (using the same pad for ∼12 hours) at baseline, causing chaffing and soreness.^3^ As the normal vaginal microbiota is protective against the acquisition of STI,^26–29^ and HIV,^30^ ^31^ menstrual cups may have the potential to reduce the incidence of such infections in vulnerable girls and the spread to their partners.

Menstrual cups are available throughout the world, with numerous brands used by many millions of girls and women, including in countries of SSA, South America and in India.^44^ While availability in rural impoverished areas is very limited, different marketing strategies among the available brands are evolving including donation of a free second cup for each cup sold. However, while advocacy is building internationally to support girls' menstrual needs in school, and many national governments are including menstrual care in their guidance, evidence on the health and educational benefits is required to further galvanise international commitment and funding.^2^

The growth of *E. coli* on cups of girls in the first 6 months could pose a hygiene risk. Poor personal hygiene, such as inadequate wiping after defaecation, occurred among prepubescent girls with vulvovaginitis in Turkey, with 10 different micro-organisms isolated including *E. coli*.^48^ Girls in our cups group received training to boil cups after each menses, they were provided with soap, and trained to wash their hands before cup emptying.^39^ However, accidental dropping of cups occurred in the first 6 months as girls had to learn to empty and reinsert their cup, which may have contributed to the excess *E. coli*.^38^ It is thus recommended that future menstrual cup programmes in schools ensure high-quality training on hygiene, and liaise with schools to advise on adequate school WASH facilities.

This study has several limitations. We chose primary schoolgirls to cover time of menarche and sexual debut; however, the introduction of free primary education has reduced the average age of girls attending primary school,^49^ and many girls had not reached the median age of sexual debut in this area (16.1 years).^50^ Thus, sexual exposure and dropout due to pregnancy or marriage were lower than expected in the control arm.^50^ Migration occurred in 10.9% of enrolled participants, which may have concealed undeclared pregnancies among girls lost to follow-up,^23^ and may have reduced the ability to detect dropout, but is unlikely to have biased our findings as there was no difference in migration rates between study arms. Furthermore, as our study was nested in a HDSS, it allowed us to visit participants' homes to confirm cases of migration or school dropout, and to document any pregnancies and related birth outcomes. This approach minimised the under-reporting of pregnancy-related dropouts due to the stigma associated with premarital sex, although some still may have been missed.

The study was designed as an exploratory feasibility study to support the design of future trials and was not powered to detect small-to-moderate differences in outcomes between study arms, especially taking the relatively short duration of follow-up into account (median 10.9 months). The study duration was compromised by a national teachers' strike at the start of enrolment, plus late entry of younger girls who could only enter the study once they reached menarche. The relatively short intervention period was compounded by the slow initial uptake of cups, requiring time for familiarisation before girls used them regularly,^37^ as reported in a Nepalese study.^51^ Our results suggest 9 or more months intervention were required before potential beneficial effects became apparent, reducing our study power as only two-thirds of girls were followed for at least 9 months, and half of them for more than 1 year. This short duration limited the study's ability to detect differences in school dropout. The rate of dropout appeared higher during early intervention, and declined in the second year for participants followed longer, which may reflect that the most vulnerable girls were among early dropouts, including those at higher risk of STI. Studies to date, predominantly using school registers, have not shown a strong association between absence and menstrual intervention with varying duration of follow-up ranging between 1-month and 1-year.^10^ ^12–16^ Attendance data may be a poor measure of menstrual intervention effectiveness in SSA where girls report multiple reasons for absenteeism.^15^ The limitations of routine registration has also been noted in school-based helminth studies.^52^ One menstrual cup study using a combination of school registers and time diary data for a subset of girls was able to ascertain that girls miss a total of 0.4 menstruating days in a 180 day school year in Nepal.^14^ We could not use our calendar data to confirm or refute this; although girls filled in the days of their menstruation, many did not self-report absence days. Lack of quantifiable school absence here and in other pilot studies,^10^ ^51^ contrasts with girls',^3–5^ ^10^ and parents narratives.^37^ It is unclear if girls in this environment exaggerate missed time when in focus groups but not in structured questionnaires,^15^ if girls avoid reporting reasons that are associated with stigma (such as teenage pregnancy), fear repercussions when reporting absence (which we consider possible in our study), or if menstrual absence due to abdominal cramps or other reasons over-ride any menstrual product effect.^15^ ^23^ Further research is needed to better quantify the effects of menstrual interventions on school absence, and it seems prudent that studies validate their measurement tools in pilot studies and spot-checks.

The impact on STIs was assessed using the prevalence of STIs at the end of the follow-up. Logistical constraints prevented testing for STIs at baseline and any pre-existing differences in STI prevalence cannot be excluded. It is recommended that future trials evaluating these interventions assess the incidence of STIs. Similarly, collection of baseline and end line sexual behaviours would confirm or refute any hypothesised intervention effect.

An important component of this study was the necessity to ensure that cup users were able to use them hygienically. Owing to the limited literature on the potential risk of poor school hygiene on menstrual cup safety, we selected schools based on pupil–latrine ratio, some separate latrines for girls, and the availability of water at the baseline spot-check. This limits the generalisability of our study, and it is unclear if the results can be extrapolated to schools without these conditions; however, while these inclusion criteria were met at baseline, observational follow-up and separate discussions with girls revealed conditions fluctuated and schools often did not supply soap or water.^37^ It is noted that concerted effort is underway in many countries including Kenya to address WASH in schools, with national guidelines on minimal standards.^53^ Adequate hygiene also required preintervention education, provision of soap throughout the study, and presence of a nurse in the schools. Soap was provided to all girls, to ensure a balance between the arms. Girls in the control group received small (non-menstrual) items, such as pencils or a biro pen, at similar intervals to those in the other arms for diary completion. This frequent exposure to study nurses was appreciated by all girls, including those in the control group, and may have positively influenced girls' attitude to school and attendance.

In conclusion, our feasibility study found that provision of menstrual cups and sanitary pads for ∼1 school-year was not associated with a significant effect on school dropout, but was associated with a lower risk of STIs in schoolgirls in western Kenya. We hypothesise this is due to reducing the need for transactional sex to obtain menstrual products. These results are relevant to other areas with similar high frequency of transactional coerced sex among adolescent girls. Our study also suggests that cup use may be associated with less bacterial vaginosis, which is common in SSA and a known risk factor for STIs and HIV acquisition and transmission. Menstrual cups are safe to use and well accepted after a familiarisation phase,^37^ are approved by the US Food and Drug Administration, and can be reused for up to 10 years, and so are more cost-effective than pads. This was a pilot study and the findings now need to be confirmed in a larger trial with longer follow-up among secondary schoolgirls, who are vulnerable to sexual coercion and pregnancy-related dropout.^23^ Metagenomic studies evaluating the effect of menstrual cups on the vaginal microbiome and subsequent potential effect on STI, HIV transmission, and maternal health are also recommended.
