# Supplementary material for: Menstrual cups and sanitary pads to reduce school attrition, and sexually transmitted and reproductive tract infections: a cluster randomised controlled feasibility study in rural Western Kenya
Source: BMJ Open. 2016 Nov 23;6(11):e013229. doi: 10.1136/bmjopen-2016-013229 (PMC5168542; doi:10.1136/bmjopen-2016-013229)
Supplement: Supplementary data [file bmjopen-2016-013229supp.pdf]

Supplementary Tables and Figures

Table S1 Proportion of participants followed-up by 9 month and 12 month cut off points

Duration	Cups (N=188)	Pads (N=256)	Control (N=200)	Total (N=644)
**Cut off at 9 months**				
0 to 9 month	64(34.0%)	80(31.3%)	74(37.0%)	218(33.9%)
9 or more months	124(66.0%)	176(68.8%)	126(63.0%)	426(66.1%)
**Cut off at 12 months**				
0 to 11 months	95(50.5%)	119(46.5%)	110(55.0%)	324(50.3%)
12 or more months	93(49.5%)	137(53.5%)	90(45.0%)	320(49.7%)

Table S2 Reason for dropping out of school

Characteristics	Cups (N=188)	Pads (N=256)	Control (N=200)	Total (N=644)
No dropout events	167	230	184	581
All dropout events	21	26	16	63
**Reported dropout events**				
Pregnancy-related	12 (57.1%)	15 (57.7%)	11 (68.8%)	38 (60.3%)
Marriage	1(4.8%)	0(0.0%)	1(6.3%)	2(3.2%)
Drop-out other reasons	7(33.3%)	9(34.6%)	3(18.8%)	19(30.2%)
Not able to identify reason	1(4.8%)	2(7.7%)	1(6.3%)	4(6.3%)

Table S3 Effect of cup use vs non-use on composite outcome~, overall, and by duration of intervention

FU‡	Prevalence (%)			Unadjusted PR (95% CI) §				Adjusted PR (95% CI) §			
	Evidence cup used †	Cup not used	Control	Cup used v not used	Cup used v control	Cup not-used v control	Cup used v not used + control	Cup used v not used	Cup users v control	Cup not used v control	Cup used v not used + control
All	13/117(11.1)	14/48(29.2)	28/172(16.3)	0.38(0.20,0.70)	0.68(0.40,1.15)	1.79(1.00,3.22)	0.51(0.31,0.83)	0.38(0.20,0.71)	0.66(0.38,1.14)	1.74(1.03,2.93)	0.50(0.30,0.84)
Stratified at 9 months follow-up											
<9m	6/32(18.8)	8/23(34.8)	8/59(13.6)	0.54(0.32,0.92)	1.38(0.60,3.20)	2.57(1.24,5.30)	0.86(0.47,1.58)	0.60(0.30,1.23)	1.49(0.61,3.67)	2.48(1.22,5.04)	0.95(0.46,1.97)
≥9m	7/85(8.2)	6/25(24.0)	20/113(17.7)	0.34(0.10,1.19)	0.47(0.25,0.86)	1.36(0.47,3.93)	0.40(0.18,0.91)	0.34(0.10,1.16)	0.48(0.25,0.91)	1.42(0.48,4.18)	0.40(0.17,0.92)
Stratified at 12 months follow-up											
<12m	11/53(20.8)	12/31(38.7)	16/93(17.2)	0.54(0.29,0.99)	1.21(0.58,2.50)	2.25(1.15,4.38)	0.80(0.45,1.44)	0.61(0.32,1.19)	1.18(0.55,2.56)	1.93(1.08,3.45)	0.85(0.44,1.65)
≥12m	2/64(3.1)	2/17(11.8)	12/79(15.2)	0.27(0.03,2.45)	0.21(0.06,0.73)	0.77(0.19,3.93)	0.23(0.05,1.18)	-	-	-	-

Abbreviations: CI: confidence interval; FU: follow-up time after intervention provision; <9m: less than 9 months; ≥9m: 9 months or greater; <12m: less than 12 months; ≥12m: 12 months or greater

~ The composite variable is a combination of dropout and laboratory confirmation of the presence or absence of a tested STI among non-dropouts at endline

§ Covariates in adjusted analysis are age, socio-economic status, and reported sexual activity (had sexual intercourse) at start of study

† Evidence based on physical observation of cup colour change at participant follow-up by study nurse.

Figure S1 - The Mooncup®

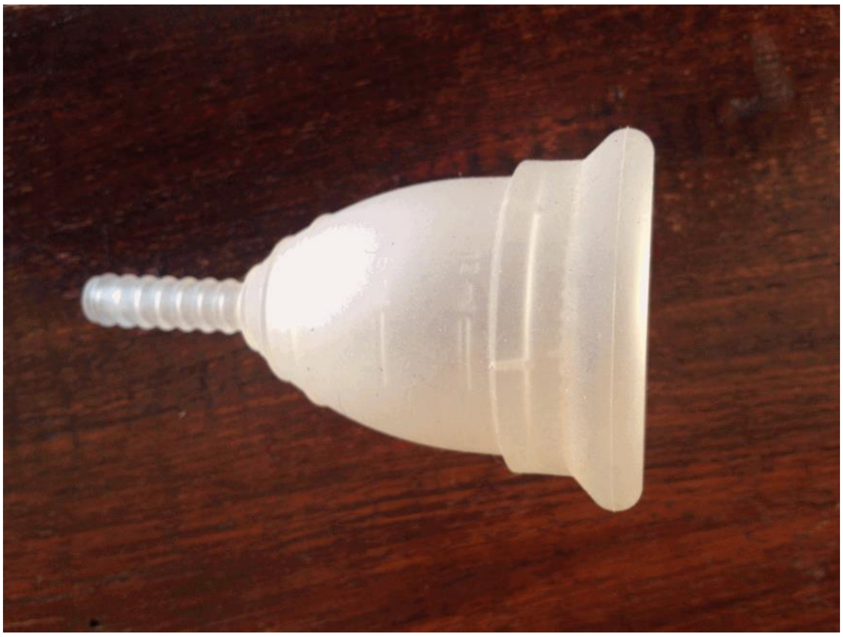

Figure S2 - Impact of pads and cups on school dropout, sexually transmitted and reproductive tract infections by 12 months duration

Prevalence/group	Comparison	Prevalence Ratio (95% CI)	*P*	Adjusted Prevalence Ratio (95% CI)§	*P*
**School dropout, <12 months follow-up**					
Cups: 20/95 (21.1%)	Cups vs. Pads	1.09 (0.50, 2.35)	.828	1.03 (0.47, 2.23)	.949
Pads: 23/119 (19.3%)	Cups vs. Control	1.93 (1.04, 3.59)	.038	1.88 (0.98, 3.62)	.058
Cups+Pads: 43/214 (20.1%)	Pads vs. Control	1.77 (0.80, 3.94)	.160	1.84 (0.83, 4.04)	.132
Control: 12/110 (10.9%)	Cups+Pads vs. Control	1.85 (1.01, 3.38)	.046	1.86 (1.01, 3.43)	.047
**School dropout, ≥12 months follow-up**					
Cups: 1/93 (1.1%)	Cups vs. Pads	0.49 (0.07, 3.35)	.468		
Pads: 3/137 (2.2%)	Cups vs. Control	0.24 (0.03, 1.80)	.166		
Cups+Pads: 4/230 (1.7%)	Pads vs. Control	0.49 (0.11, 2.19)	.352		
Control: 4/90 (4.4%)	Cups+Pads vs. Control	0.35 (0.08, 1.53)	.161		
**Combined STIs*, <12 months follow-up**					
Cups: 3/64 (4.7%)	Cups vs. Pads	1.36 (0.43, 4.30)	.601		
Pads: 3/87 (3.4%)	Cups vs. Control	0.95 (0.26, 3.41)	.936		
Cups+Pads: 6/151 (4.0%)	Pads vs. Control	0.70 (0.21, 2.29)	.553		
Control: 4/90 (4.4%)	Cups+Pads vs. Control	0.81 (0.27, 2.42)	.712		
**Combined STIs*, ≥12 months follow up**					
Cups: 3/80 (3.8%)	Cups vs. Pads	0.72 (0.27, 1.92)	.510	0.67 (0.26, 1.72)	.401
Pads: 6/115 (5.2%)	Cups vs. Control	0.35 (0.13, 0.92)	.033	0.32 (0.13, 0.79)	.014
Cups+Pads: 9/195 (4.6%)	Pads vs. Control	0.49 (0.24, 1.00)	.049	0.48 (0.25, 0.93)	.029
Control: 8/75 (10.7%)	Cups+Pads vs. Control	0.41 (0.21, 0.83)	.012	0.39 (0.21, 0.74)	.004
**Combined RTIs**, <12 months follow up**					
Cups: 13/64 (20.3%)	Cups vs. Pads	0.68 (0.41, 1.13)	.136	0.64 (0.38, 1.08)	.094
Pads: 26/87 (29.9%)	Cups vs. Control	0.69 (0.39, 1.20)	.189	0.68 (0.40, 1.16)	.155
Cups+Pads: 39/151 (25.8%)	Pads vs. Control	1.01 (0.62, 1.64)	.972	1.07 (0.68, 1.67)	.780
Control: 24/81 (29.6%)	Cups+Pads vs. Control	0.83 (0.52, 1.32)	.433	0.85 (0.56, 1.29)	.447
**Combined RTIs**, ≥12 months follow up**					
Cups: 18/80 (22.5%)	Cups vs. Pads	0.81 (0.46, 1.41)	.453	0.80 (0.47, 1.38)	.427
Pads: 32/115 (27.8%)	Cups vs. Control	0.94 (0.49, 1.80)	.847	0.92 (0.48, 1.74)	.787
Cups+Pads: 50/195 (25.6%)	Pads vs. Control	1.16 (0.74, 1.81)	.515	1.14 (0.74, 1.75)	.547
Control: 18/75 (24.0%)	Cups+Pads vs. Control	1.04 (0.64, 1.70)	.867	1.02 (0.64, 1.64)	.929
Bacterial Vaginosis, <12 months follow up					
Cups: 12/64 (18.8%)	Cups vs. Pads	0.91 (0.53, 1.55)	.720	0.89 (0.53, 1.52)	.683
Pads: 18/87 (20.7%)	Cups vs. Control	0.84 (0.50, 1.42)	.525	0.87 (0.50, 1.50)	.611
Cups+Pads: 40/151 (26.5%)	Pads vs. Control	0.93 (0.55, 1.57)	.789	0.97 (0.57, 1.67)	.911
Control: 18/81 (22.2%)	Cups+Pads vs. Control	0.89 (0.57, 1.39)	.599	0.92 (0.58, 1.46)	.720
Bacterial Vaginosis, ≥12 months follow up					
Cups: 9/80 (11.3%)	Cups vs. Pads	0.59 (0.34, 1.03)	.064	0.60 (0.33, 1.08)	.091
Pads: 22/115 (19.1%)	Cups vs. Control	0.60 (0.33, 1.08)	.091	0.60 (0.32, 1.11)	.102
Cups+Pads: 31/195 (15.9%)	Pads vs. Control	1.02 (0.64, 1.65)	.920	1.00 (0.61, 1.62)	.986
Control: 14/75 (18.7%)	Cups+Pads vs. Control	0.79 (0.50, 1.24)	.300	0.77 (0.48, 1.23)	.280

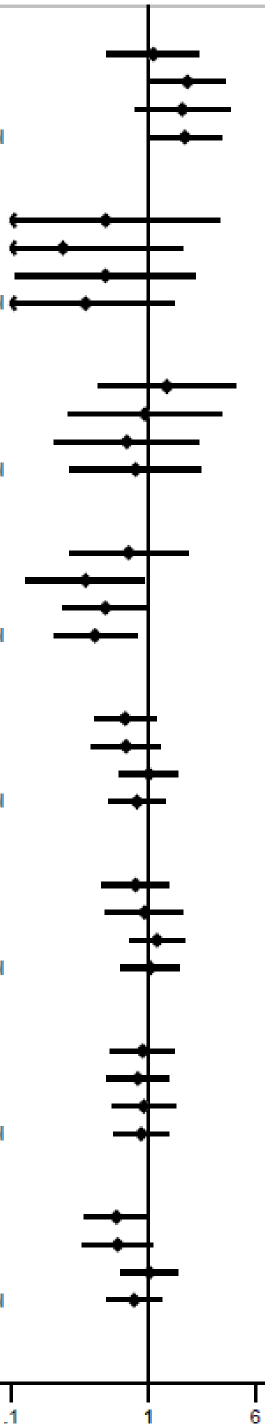

STI: sexually transmitted infections; RTI: reproductive tract infections; § Adjusted for age, socio-economic status, and reported sexual activity (had sexual intercourse) at start of study.
* Composite of STIs at endline (presence of either *Chlamydia trachomatis, Trichomonas vaginalis, or Neisseria gonorrhoea*); ** Composite RTIs tested at endline (presence of either bacterial vaginosis or *Candida albicans*).
